# The impact of reconstructed soils following oil sands exploitation on aspen and its associated belowground microbiome

**DOI:** 10.1038/s41598-018-20783-6

**Published:** 2018-02-09

**Authors:** Franck Stefani, Nathalie Isabel, Marie-Josée Morency, Manuel Lamothe, Simon Nadeau, Denis Lachance, Edith H. Y. Li, Charles Greer, Étienne Yergeau, Bradley D. Pinno, Armand Séguin

**Affiliations:** 1 Natural Resources Canada, Canadian Forest Service, Laurentian Forestry Centre, Québec, G1V4C7 Canada; 2 Agriculture and Agri-Food Canada, Ottawa, K1A 0C6 Canada; 3 National Research Council Canada, Energy, Mining and Environment, Montréal, H4P 2R2 Canada; 4 Institut national de la recherche scientifique, Centre INRS-Institut Armand-Frappier, Laval, H7V 1B7 Canada; 5 Natural Resources Canada, Canadian Forest Service, Northern Forestry Centre, Edmonton, T6H 3S5 Canada

## Abstract

The objective of this study was to investigate the impact of different soil covers used to reclaim decommissioned oil sands mining sites on the genetic diversity of aspen and their associated belowground microbiota. Aspen genotyping showed that trees mostly originated from sexual reproduction on sites reclaimed with soil covers made of upland forest floor-mineral mix (FFMM) and lowland peat-mineral mix (PMM). In contrast, most individuals in mature and burned stands sampled as benchmarks for natural disturbances originated from vegetative reproduction. Nonetheless, aspen populations in the FFMM and PMM sites were not genetically different from those in mature and burned stands. DNA metabarcoding of bacteria and fungi in root and soil samples revealed that the diversity of the belowground microbiota associated with aspen and the relative abundance of putative symbiotic taxa in PMM were significantly lower than for FFMM and naturally disturbed sites. Despite similar aspen genetic diversity between FFMM and PMM sites, trees were not associated with the same belowground microbiota. Because the soil microbiome and more specifically the mycorrhizal communities are variable both in space and time, long-term monitoring is particularly important to better understand the ecological trajectory of these novel ecosystems.

## Introduction

Oil sands surface mining in the Athabasca region of northern Alberta (Canada) is a significant anthropogenic disturbance that resets boreal forest development and succession to early stages. During surface mining, the vegetation is cleared, the topsoil is salvaged, and the overburden (the layer of sand and clay in between the topsoil and the oil sands) is piled (see Fig. 2 in Audet *et al*.^1^ for an explanation of the land-reclamation procedure). After mining is finished, the landforms (composed of overburden and tailings sand) are capped with organic matter-rich soil covers made of either upland-derived forest floor-mineral mix (FFMM) or a lowland-derived peat-mineral mix (PMM)^2^. Alberta’s oil sands deposits represent an economically viable proven reserve of about 166 billion barrels of crude bitumen covering 142,200 km^2^ of land in the boreal forest^3^. Among the three oil sands deposits in Alberta (Peace River, Cold Lake and Athabasca areas), surface mining is only possible in the Athabasca region. The surface mineable area represents a total of 4,750 km^2^, with 895 km^2^ of land already disturbed^4^. Ecosystems affected by oil sands surface mining lose their ecological functions due to the disappearance of above- and belowground species^5^. It is therefore crucial to reclaim these areas using strategies that provide the best conditions for site recolonisation in order to quickly reestablish a self-sustaining and functioning ecosystem. Reclamation is the process of recovering ecosystem services through revegetation but not necessarily with the original species^6^. Land reclamation in Alberta is based on the concept of equivalent land capability, which requires that the disturbed site be returned to conditions that support various land uses similar to pre-disturbance conditions though not necessarily identical^7^, thus leading to the emergence of new ecosystems. Sites under reclamation represent a unique opportunity to study the plant and microbial colonization of new ecosystems.

Aspen (*Populus tremuloides* Michx.) naturally colonises the sites under reclamation^8^, but its genetic diversity and origin is unknown. The development and health of trees rely on their interactions with the soil microbiome^9^. Short roots of most trees in boreal and temperate forests are colonised by mycorrhizal fungi that greatly facilitate access to nutrients^10,11^, and with fungal endophytes whose ecological roles are unclear^12^. For instance, Horton *et al*.^13^ showed in Californian pine forests that seedlings were colonised with the resident inoculum within the first days following establishment and the entire root system was colonised after six months, mostly by ectomycorrhizal fungi, and to a lesser extent, by root fungal associates. This quick connection to the soil network of fungal symbionts and its associated bacterial communities^14–16^ is key for nutrient acquisition and plant productivity^17^. Moreover, the presence of such a microbial inoculum in the soil cover could speed up the return to a functioning ecosystem state in reclaimed areas.

Currently, there are considerable knowledge gaps in bacterial and fungal communities present in the different types of soil cover used in oil sands mine reclamation or other industrial sites. The qualitative and quantitative differences in microbiome diversity between each soil cover (i.e. FFMM vs PMM) are unknown, and it has yet to be determined how they compare with the soil microbiomes from naturally disturbed sites. Previous studies that investigated the environmental performance of different reclamation treatments focused on the physical, chemical and biological properties of these artificial soils and monitored the soil microbial communities using fingerprinting methods such as denaturing gradient gel electrophoresis (DGGE) and phospholipid fatty acid (PLFA)^18–22^.

To better understand the ability of the different reclamation strategies to return heavily disturbed industrial sites in a state of functional ecosystems, the aboveground host genetic and belowground bacterial and fungal diversity in sites reclaimed with FFMM and PMM soil covers were characterized and compared to ancient (mature aspen stands) and recent (three-year-old burned aspen stands) naturally disturbed sites (hereafter Mature and Fire sites). More specifically, we addressed the following hypotheses: (i) Aspen in reclaimed sites are genetically different from those in the surrounding naturally disturbed stands, (ii) Different types of soil cover result in differences in taxonomic and putative functional diversities of aspen root and soil microbiomes, and (iii) Aspen root and soil microbiomes from the different types of soil cover are different from those in naturally disturbed stands.

## Results

### Genetic diversity of aspen

More than 11,800 high quality variants from the genotyping by sequencing GBS data were used to estimate genetic diversity parameters for aspen in reclaimed and natural sites. A very high degree of clonality was detected among sampled *P. tremuloides* trees from the Mature and Fire sites, while fewer clones were observed in the FFMM and PMM sites (Fisher’s exact test: *p* = 2.2 × 10^−16^ between natural and reclaimed sites) (Table 1). The proportion of unique genotypes was lower (*p* = 0.04) among three-year-old post-fire stands (clonal richness R = 0.08) than Mature stands (R = 0.20), while the FFMM and PMM sites were not significantly different and included together 96% of unique genotypes. No identical clones were detected between adjacent stands.

**Table 1:** Clonal richness (R) of aspen found in the peat-mineral mix (PMM), forest floor-mineral mix (FFMM), Mature and Fire sites.

Sites	Stands	No. of trees sampled	No. of unique genotypes	R	Weighted R per site	Weighted R per site type
*Reclaimed*						
PMM	1	30	28	0.93	0.95	0.96
	2	28	27	0.96		
	3	13	13	1		
	4	27	25	0.92		
FFMM	1	24	23	0.96	0.98	
	2	4	4	1		
	3	10	10	1		
	4	14	14	1		
*Natural*						
Fire	1	28	2	0.04	0.08	0.12
	2	29	3	0.07		
	3	25	3	0.08		
	4	27	4	0.12		
Mature	1	6	3	0.40	0.20	
	2	22	4	0.14		
	3	19	3	0.11		
	4	14	5	0.31		

**Figure 1:**
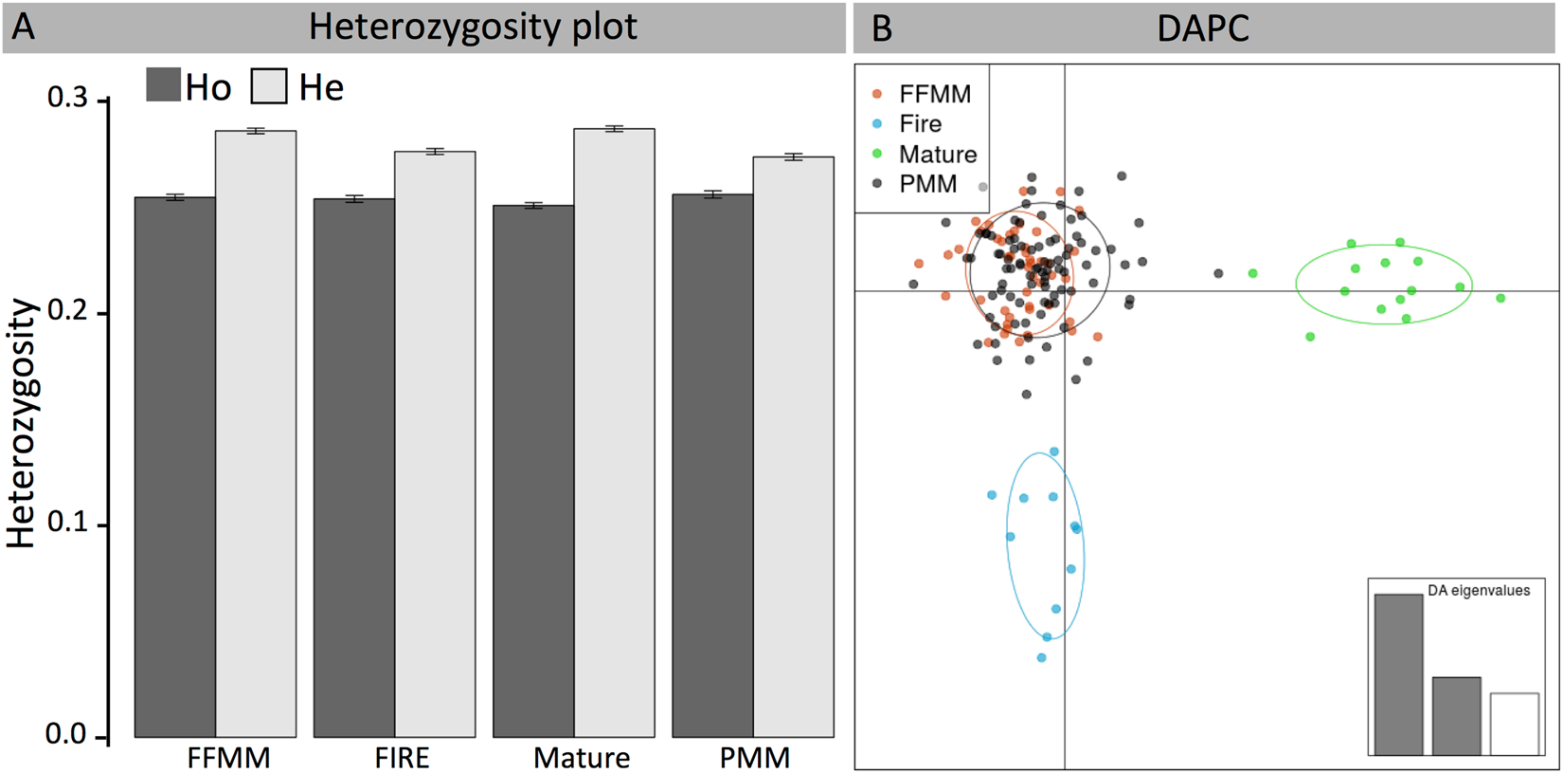
(**A**) Observed (H_O_) and expected (H_E_) heterozygosity of aspen in the PMM (peat-mineral mix), FFMM (forest floor-mineral mix), Mature and Fire sites. Error bars represent the standard errors. (**B**) Biplot of the first two axes of a discriminant analysis of principal components (DAPC) on aspen GBS data using the four types of sites (PMM, FFMM, Mature and Fire) as groups. The inset shows the eigenvalues of each discriminant axis (total of 33% of the genetic variance explained).

Genetic diversity estimates, as evaluated by observed (*H*_O_) and expected heterozygosity (*H*_E_) did not significantly differ between FFMM, PMM, Mature, and Fire sites (Fig. 1A; *H*_O_: *P* = 0.254; *H*_E_: *P* = 0.280). For all four types of sites, *H*_O_ was slightly lower than *H*_E_. The estimated global *F*_ST_ was close to zero (*F*_ST_ = 0.003) and Nei’s pairwise *F*_ST_ values among sites were the largest between Mature and Fire natural sites (*F*_ST_ = 0.027) and the lowest between the FFMM and PMM reclaimed sites (*F*_ST_ = 0.005) (Table 2). DAPC *k*-means analyses found that only one genetic cluster (*k* = 1) best explained the data, as it showed the lowest BIC value. However, in order to describe the population differentiation between FFMM, PMM, Mature and Fire sites, we performed a DAPC analysis using the four types of sites as pre-defined groups. A biplot of the first two discriminant functions (totaling 36% and 20% of the total genetic variation, respectively) showed that Fire and Mature sites are slightly differentiated from other *P. tremuloides* samples (Fig. 1B).

**Table 2:** Pairwise F_ST_ statistics among sites for aspen.

Sites	PMM	FFMM	Fire	Mature
*Reclaimed*				
PMM	—	0.004	0.007	0.007
FFMM	0.004	—	0.011	0.012
*Natural*				
Fire	0.007	0.011	—	0.024
Mature	0.007	0.012	0.024	—

### Limited belowground microbial diversity in peat-mineral mix soil cover

**Figure 2:**
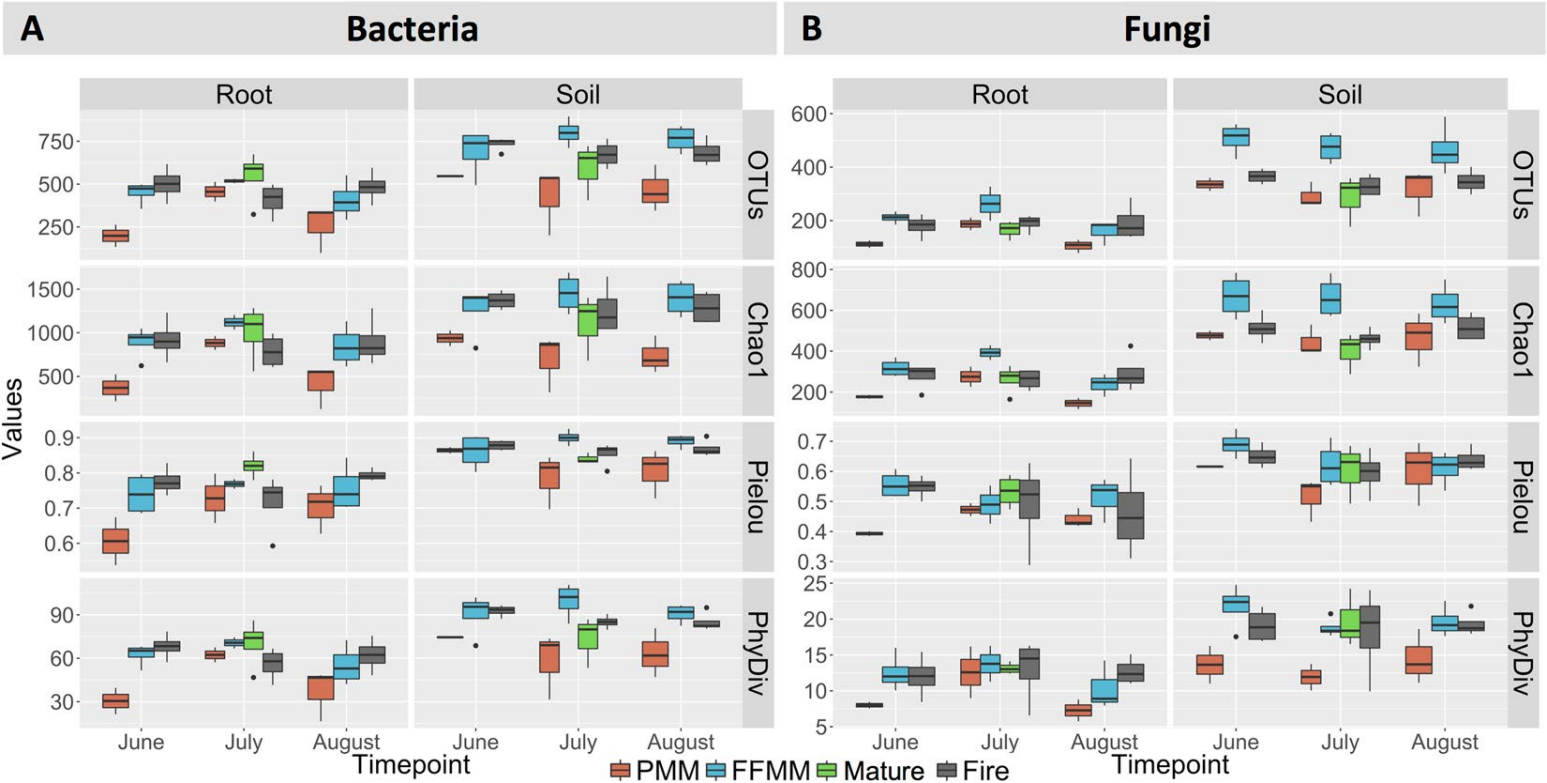
Alpha diversity metrics calculated for (**A**) bacterial and (**B**) fungal communities recorded in soil and root samples collected in June, July and August 2014 at the PMM, FFMM, Fire and Mature sites. OTUs represent the number of observed OTUs; the chao1 richness estimator was calculated using the bias-corrected version of the equation; the metrics Pielou and PhyDiv are Pielou’s evenness and Faith’s Phylogenetic diversity indexes, respectively. For the fungal community, PhyDiv was calculated on the phylogenetic tree that only includes OTUs identified as Agaricomycetes. Error bars represent 95% confidence interval around the mean.

The analysis of variance performed on four diversity indices calculated for the bacterial and fungal communities recorded in root and soil samples (Fig. 2) indicated that sampling time had no significant impact (Bonferroni correction *P* > 0.025). For the root samples, no significant differences were observed in the diversity metrics calculated from the PMM, FFMM and Fire sites for the bacterial and fungal communities. For the soil samples, the diversity metrics calculated for the bacterial community in PMM were significantly lower (Bonferroni correction *P* < 0.025) compared to the FFMM and Fire sites (Fig. 2). The diversity metrics calculated for the bacterial and fungal community in soil and root samples from the Mature site had values comparable to those from the FFMM and Fire sites. The soil fungal community recorded in the FFMM site had a greater diversity (*P* < 0.001) when compared to the Fire and PMM sites, although the phylogenetic diversity index calculated for Agaricomycetes was similar to that recorded in Fire and Mature sites. Soil fungal OTUs richness in the PMM site was comparable to that in Mature and Fire sites, but the phylogenetic diversity of the Agaricomycetes community recorded in PMM was significantly lower than that recorded in the FFMM (*P* = 9.7e-05) and Fire (*P* = 0.001) sites.

**Figure 3:**
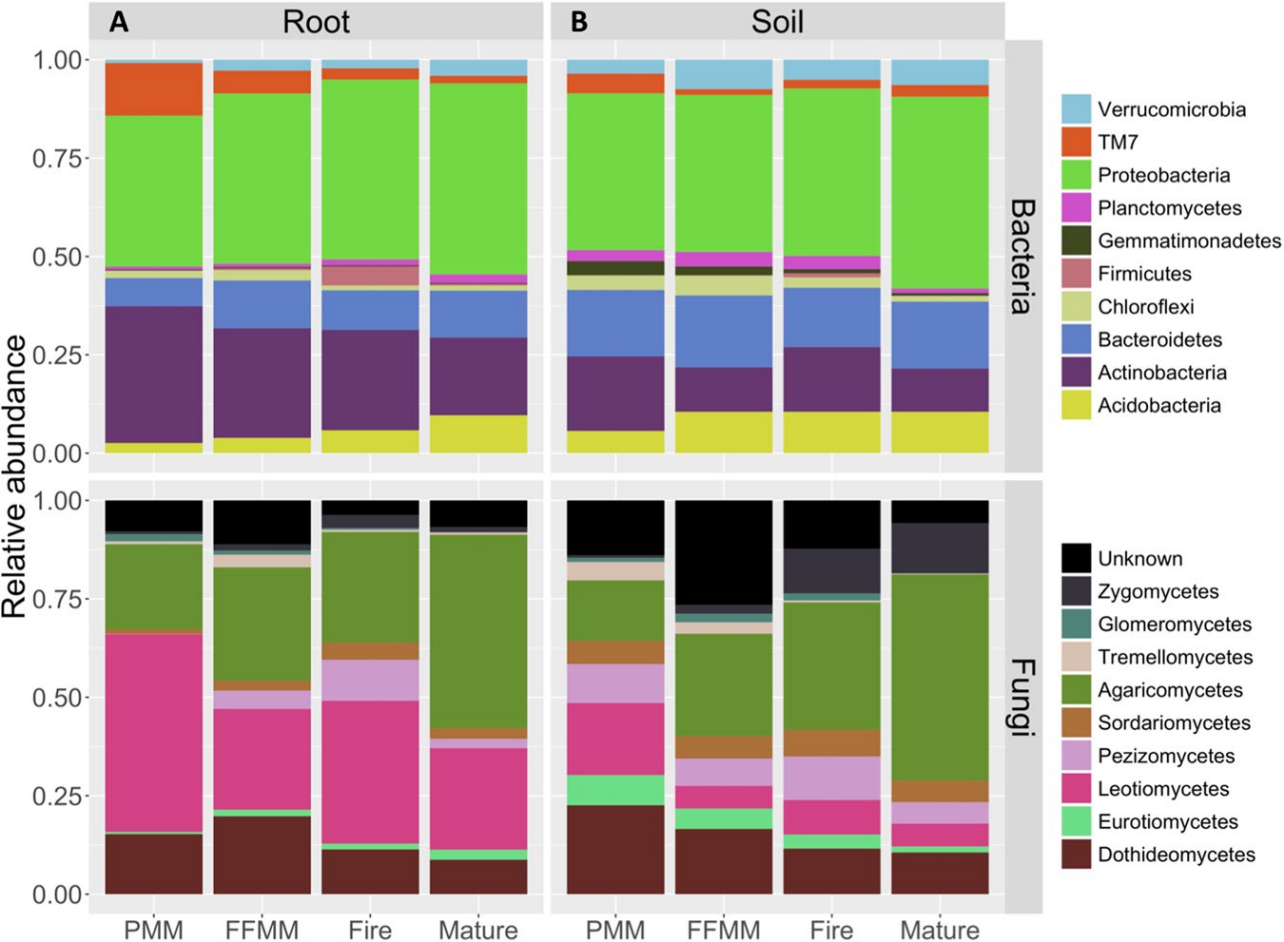
Taxonomic profile of the bacterial and fungal communities recovered in (**A**) root and (**B**) soil samples from the PMM, FFMM, Fire, and Mature sites. Bacterial phyla and fungal classes with a relative abundance <1% are not shown. Data represents the number of reads of a taxonomic group.

The relative abundance of the most abundant bacterial phyla observed in aspen root samples gradually changed from PMM/FFMM to Fire and Mature sites (Fig. 3). For instance, the relative abundance of *Acidobacteria* was 2.6%, 3.9%, 5.8% and 9.6% in PMM, FFMM, Fire and Mature sites, respectively. The relative abundance of *Actinobacteria* varied differently, with a relative abundance ranging from 34.8% in PMM to 19.7% in Mature site. Similarly, the relative abundance of the candidate division TM7 observed in root samples decreased from 13.4% in PMM to 2% in the Mature site. The taxonomic profiles at the phylum level recorded in the soil samples from the four types of sites were broadly similar apart from the relative abundance of *Acidobacteria* which was 5.6% in PMM compared to 10.5% on average in the other sites. The analyses of the fungal taxonomic profiles (Fig. 3) in aspen root samples showed that Letiomycetes dominated the fungal community in the PMM site (50% of relative abundance compared to 29.2% on average in other sites). In contrast, the relative abundance of Agaricomycetes was 21.6% in PMM compared to 49.1% in aspen roots from the Mature site. This difference in the proportion of Agaricomycetes followed the same trend in soil samples as well (15.3% in PMM, 52.4% in Mature sites). Taxa belonging to the Zygomycetes were uncommon in PMM and FFMM sites compared to what was found in Mature and Fire sites.

### Different microbial community structures and functional profiles in peat-mineral mix soil cover

**Figure 4:**
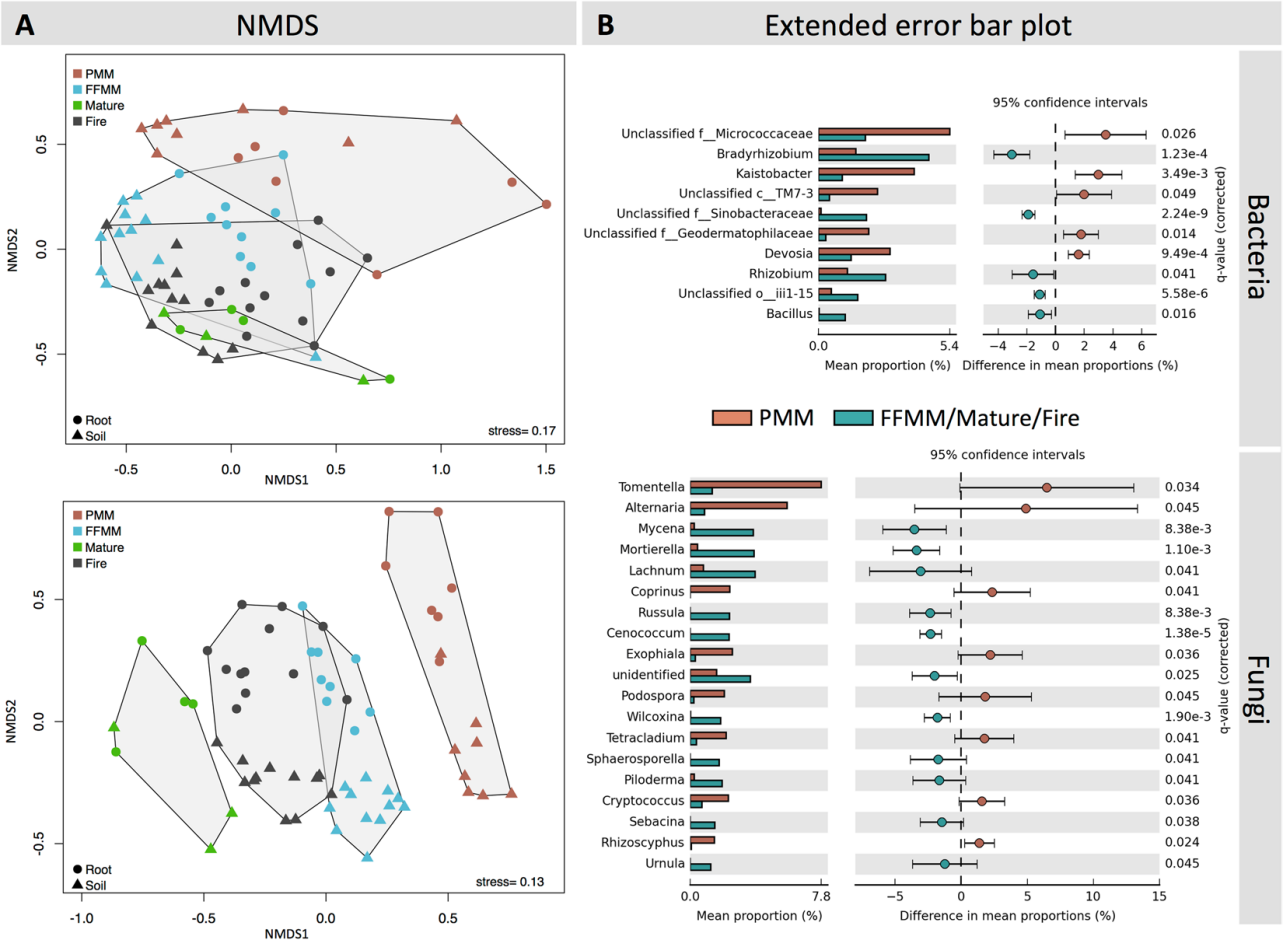
(**A**) Nonmetric multidimensional scaling of the bacterial (top row of figures) and fungal (bottom row of figures) communities recorded in soil and root samples, performed at the class level between the four sites and (**B**) extended error bar plots of the genera which abundances are significantly different (P < 0.05 and effect size >1) between PMM and FFMM/Mature/Fire. Bacterial and fungal genera are sorted by effect size. The 95% confidence intervals were calculated using Welch’s t-test. Bar plots represent the mean abundance of the reads in each pair of groups (PMM with FFMM/Mature/Fire).

Based on NMDS ordinations (Fig. 4A), soil and aspen root samples from the PMM site tended to cluster distinctively from the three other types of sites, and this was more marked for fungi than for bacteria. The fungal community recorded in Mature sites was also dissimilar from the fungal community observed in Fire, FFMM and PMM. anosim R statistic was the highest when comparing the fungal community structure in PMM with Mature (*R* = 0.899, *P* = 0.0001, Table 3). For bacteria, anosim R statistic was the highest when comparing PMM with Fire (*R* = 0.699, *P* = 0.0001), followed by the comparison between PMM and Mature (*R* = 0.596, *P* = 0.0001).

**Table 3:** ANOSIM pairwise comparison on bacterial and fungal community structures from PMM, FFMM, Fire and Mature sites.

	Bacteria		Fungi	
	*R*	*P*	*R*	*P*
Fire, Mature	0.085	0.0195	0.473	0.0001
Fire, FFMM	0.372	0.0001	0.63	0.0001
FFMM, Mature	0.434	0.0003	0.84	0.0001
FFMM, PMM	0.482	0.0001	0.646	0.0001
PMM, Mature	0.596	0.0001	0.899	0.0001
Fire, PMM	0.699	0.0001	0.798	0.0001

Pairwise comparisons between sites are sorted according to the R values calculated for the bacterial community.

The extended error bar plots (Fig. 4B) indicate that the microbiome structure recorded in the PMM site was characterised by 10 bacterial OTUs and 19 fungal OTUs, with statistical evidence (*p* < 0.05 and effect size >1) that they were either increased or reduced in the PMM site compared with FFMM/Fire/Mature sites. Among the bacterial OTUs, OTUs belonging to the genera *Bradyrhizobium* and *Rhizobium* were less abundant in both root and soil samples from the PMM site, while OTUs related to the *Micrococcaceae* and the *Kaistobacter* were increased. Fungal OTUs identified as belonging to the genera *Tomentella* and *Alternaria* were significantly more abundant in the PMM site, while OTUs related to *Mycena*, *Mortierella* and *Lachnum* were poorly represented in PMM compared with the three other treatments.

**Figure 5:**
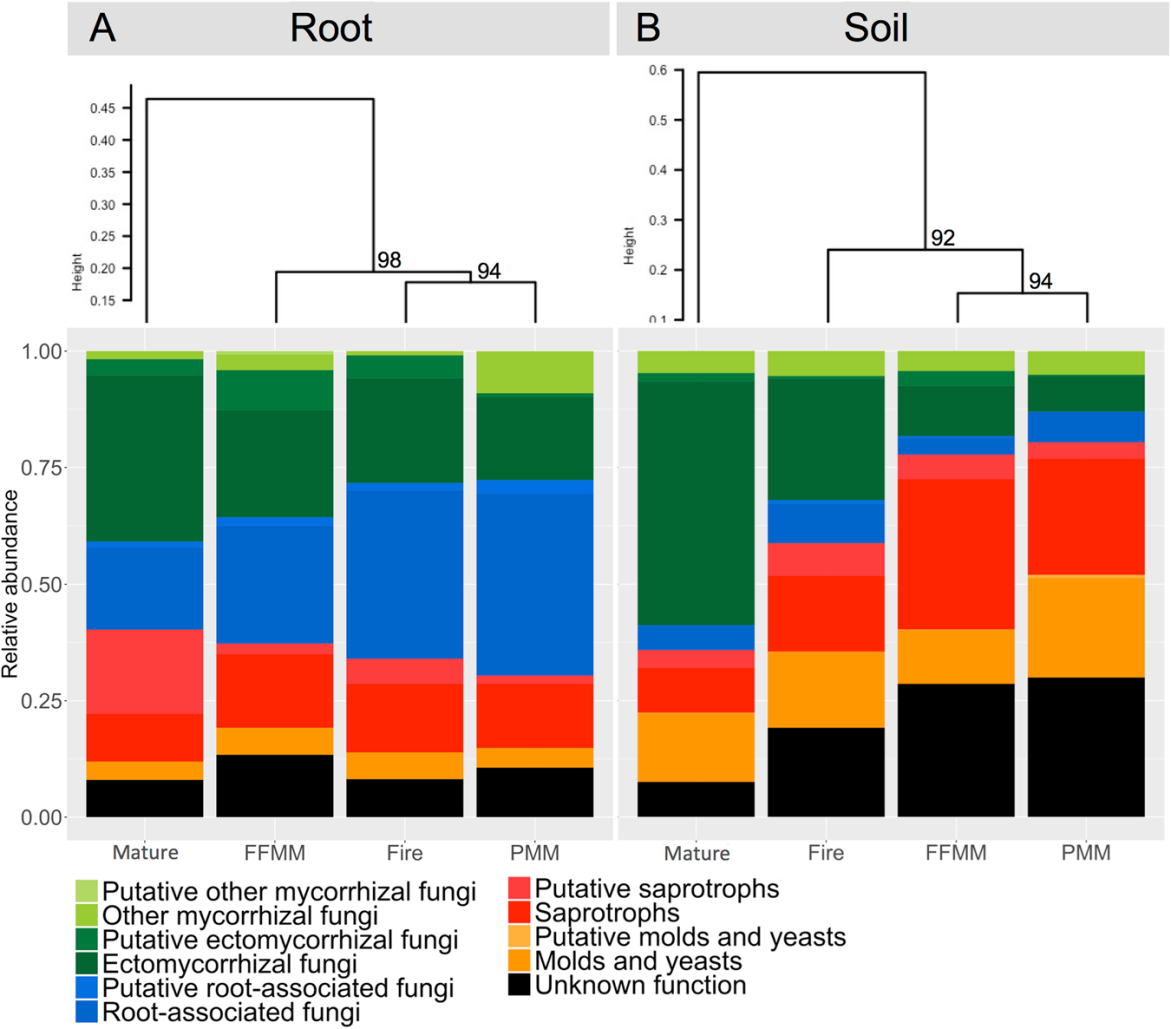
Ecological functions predicted for the fungal communities recorded in (**A**) root and (**B**) soil samples from the PMM, FFMM, Fire, and Mature sites. Sites are clustered according to the similarity of their functional profile. Values above nodes represent AU (approximately unbiased) P-values.

The fungal functional profile in the PMM site was different from the functional profile observed in the Mature sites (Fig. 5), which is in line with the analysis of the taxonomic profiles (Figs 2, 3 and 4). The mycorrhizal inoculum in PMM soil samples represented by the categories “putative other mycorrhizal fungi”, “other mycorrhizal fungi”, “putative ectomycorrhizal fungi” and “ectomycorrhizal fungi” was very limited compared to that in the Mature site. This difference could be observed in aspen root samples as well, although it was less marked. The functional profile of the fungal community in root samples from the PMM site was akin to that of the Fire site as they clustered together, while in soil samples it was comparable to that of the FFMM site. The relative abundance of mycorrhizal fungi recorded in aspen roots from PMM was 27.6% (i.e. by regrouping the Ectomycorrhizal fungi, Putative ectomycorrhizal fungi, Other mycorrhizal fungi, and Putative other mycorrhizal fungi). Interestingly, while the relative abundance of “root-associated fungi” was similar in soil samples from each site, this group was the most abundant with 40% in PMM and Fire root samples. The relative abundance of fungal taxa with unknown ecological function was higher in recently disturbed sites (PMM, FFMM and Fire) compared with the Mature site. The relative abundance of saprotrophs and putative saprotrophs in root samples was relatively similar between each site while it was higher in recently disturbed sites compared to the Mature site in soil samples. Finally, the relative abundance of taxa identified as molds and yeasts was comparable between each site for both root and soil samples.

## Discussion

Aspen genotypes and the α- and β-diversity of its belowground microbiome were determined for two reclaimed sites and two naturally disturbed sites. Results showed that (i) aspen colonised the reclaimed sites via seed recruitment rather than from vegetative propagation as in naturally disturbed sites, (ii) aspen in reclaimed sites were genetically similar to those in the surrounding natural sites, (iii) and despite the fact that aspen genetic diversity was very similar between FFMM and PMM sites, trees were not associated with the same belowground microbiota.

Based on more than 11,800 variant loci, comparative genetic analyses of aspen from the different sites revealed that vegetative propagation was the predominant mode of reproduction in old (Mature) and recent (Fire) naturally disturbed sites, with the lowest values of clonal richness observed in the Fire site (average R = 8%), followed by the Mature site (average R = 20%). Vegetative propagation is considered as the predominant mode of reproduction for this species^23,24^, and populations composed of a few clones of varying sizes were expected in natural sites^25,26^. The results obtained in this study are in line with studies conducted in aspen natural stands where the spatial distribution after natural disturbances evolve from a structured pattern (few genotypes with multiple ramets) to a more random one^27,28^. In fact, natural disturbances such as fire barely provide the windows of opportunity (seed availability, soil moisture, no competition) that would allow aspen seed to germinate^29^. In contrast to naturally disturbed sites, trees sampled on the FFMM and PMM sites three years after reclamation were primarily composed of distinct genotypes. This strongly suggests that both sites, which are surrounded by sexually mature populations of aspen, were primarily colonised via seed recruitment. The post-mining landscape reconstructed with either FFMM or PMM soil covers seems to offer adequate conditions for seedling establishment although long-term monitoring is needed to follow the ecological trajectory of these novel ecosystems^1^. The levels of genetic diversity (*H*_O_ and *H*_E_) of aspen in the FFMM and PMM sites were almost identical to those in naturally disturbed sites (Fire and Mature). Indeed, the level of genetic differentiation among all sites was very low (*F*_ST_ < 0.005) and the discriminant analysis could not distinguish FFMM from PMM. Thus, the main distinction between aspen sampled from the reclaimed and natural sites was the presence of a higher proportion of unique genotypes per sampled area. Seed recruitment following sexual reproduction, which creates new allelic combinations, might help aspen adapt to the new prevalent environmental conditions in reclaimed areas^30–32^.

Even though similar levels of genetic diversity and low genetic differentiation were observed for aspen between reclaimed and naturally disturbed sites, the belowground microbiome of aspen growing in the PMM site had less diverse microbial communities with different putative ecological functional groups of fungi compared with the three other sites. Previous studies based either on molecular data^20,33^ or on biochemical data^19,34^ also reported distinct and less diverse microbial profiles and lower microbial biomass in PMM. In the longer term, reclaimed sites were shown to be characterised by a reduced microbial biomass compared with natural sites, even several years following reclamation^35,36^. These previous studies also showed that the FFMM microbial community was more similar to undisturbed natural habitats than that from PMM soil cover. Here, we observed that the diversity and structure of the bacterial and fungal communities in FFMM were more similar to those observed in the naturally disturbed Fire site than in the Mature site.

*Proteobacteria*, *Actinobacteria* and *Bacteroidetes* were the most abundant bacterial phyla in root and soil samples with similar abundances in soil across the four sites. Masse *et al*.^37,38^ noted the same observation when they investigated the soil and root microbiomes in oil sands reclamation covers in the Athabasca Oil Sands Region. Interestingly, Masse *et al*.^37^ positively correlated the abundance of these three phyla with the estimated nitrogen deposition. This could explain why each phyla had a relatively constant abundance in the soil samples across the four sampled sites which were in the same geographical region (Supplementary Fig. S1).

Analysis of the difference in the mean proportion of bacterial genera between PMM and the three other types of sites (Fig. 4) highlighted the underrepresentation of the genera *Bradyrhizobium* sp. and *Rhizobium* sp. in PMM. Species of *Bradyrhizobium* are well known as symbiotic fixers of nitrogen^39^, but VanInsberghe *et al*.^40^ have shown that non-symbiotic living *Bradyrhizobium* ecotypes dominate coniferous forest soils across North America. While *nod* and *nif* genes are absent within the genome of these strains, ligninolytic genes are present^41^ and this metabolic activity could explain their widespread abundance and their higher gene transcription levels in forest soil^42^. Nevertheless, Bradyrhizobium-like organisms have been found as dominant taxa in the rhizosphere of *P. deltoides*^43^ and N_2_ fixation has been demonstrated in *P. trichocarpa* through diazotrophic endophytic taxa, some of them being related to the genus *Bradyrhizobium*^44^. Root endophytes belonging to the genus *Rhizobium* have been isolated in *P. deltoides*, but similarly to the free-living *Bradyrhizobium* taxa recorded in forest soil, the genes for nitrogen fixation were not found in the genome^45^. To our knowledge, the presence of nitrogen fixing rhizobia in the bulk soil and endosphere of *P. tremuloides* is not documented and it is not possible to discriminate between endosymbiotic nitrogen fixers and their free-living counterparts based on the 16S rRNA gene^40^.

*Devosia* sp. and *Kaistobacter* sp. were significantly more abundant in PMM. The genus *Devosia* is a recently described lineage outside the clade *Rhizobia* that contains nitrogen-fixing legume symbionts^46^ and, considering that boreal peatlands are water saturated much of the time, it is interesting to note that some *Devosia* strains develop root-nodule symbiosis with aquatic legume plants^47,48^. However, lowland PMM soil used in reclamation is generally placed in upland position^49^, and it is possible that this new soil environment will not be compatible with *Devosia*. Regarding *Kaistobacter* sp., it was reported from methane-enriched cultures^50^ and from diesel-contaminated arctic soils^51^, but it has also recently been found as the most abundant genus associated with suppression of the tobacco wilt disease^52^. Despite the very high total nitrogen content in PMM soil cover^53^, the bioavailability of inorganic nitrogen is limited^54^ because of the recalcitrant organic material in peat that mineralizes slowly. Nitrogen-fixing bacteria, along with mycorrhizal fungi, annually transfer to plants up to 80% of phosphorus and nitrogen in temperate and boreal forests^17,55,56^. Although the ecological function of the taxa related to *Bradyrhizobium* and *Rhizobium* recorded in this study cannot be determined, the significantly lower abundance of OTUs belonging to these genera and the low availability of nitrogen in PMM raise the question if the sites capped with this type of soil cover have sufficient fertility to sustain a switch back to the original plant community. The relationships between nitrogen-fixing bacteria and ectomycorrhizae are increasingly documented^14,57^. For instance, ectomycorrhizal root tips colonised with *Piloderma* spp. were preferentially colonised with *Rhizobiales*, such as *Bradyrhizobium*^16,57^. In the present study, the bacterial genera *Bradyrhizobium* and *Rhizobium* and the fungal genus *Piloderma* were significantly less abundant in PMM than in the three other types of sites, suggesting a similar relationship between these groups.

To our knowledge, this study also provides the first detailed view of the diversity and structure of the fungal community in oil sands sites reclaimed using different prescriptions. The soil fungal richness was higher in FFMM than in the three other types of sites due to the presence of more saprotrophs and undescribed fungi. In contrast to what was observed with bacteria, the soil fungal species richness in PMM was similar to what was recorded in the Fire and Mature sites. Despite different levels of fungal OTU richness in soil samples, the fungal functional profile in PMM and FFMM were very similar. Interestingly, the abundance of saprotrophs and taxa with unknown functions were more abundant in FFMM and PMM sites, which emphasizes the importance of sampling disturbed sites to describe fungal taxa potentially new to science.

The lower proportion of mycorrhizae in soil samples from FFMM could be attributed to soil stockpiling. The detrimental effects of this soil storage strategy have already been shown for arbuscular mycorrhizal fungi^58^ and bacteria^59^. However, PMM and FFMM in our study were directly transferred to landforms ready to be reclaimed to avoid these potential deleterious effects of stockpiling on the soil microbiome. Thus, the important decrease in the relative abundance of mycorrhizal taxa in soil samples from FFMM suggests that the operations involved in direct placement of the soil cover (forest harvest, topsoil salvage and transfer), plus the time laps for trees to reestablish and for litter to accumulate, still negatively impact soil fungi. As a consequence, the soil functional profile in FFMM resembled that of PMM. It is known that fungi are more sensitive to soil disturbance than bacteria^60,61^, mostly because of the strong dependence of symbiotic fungi upon their hosts and specialization of the saprobes involved in litter decomposition. However, the functional groups recorded in roots from FFMM shows that there remain viable ectomycorrhizal propagules to be recruited by trees.

Among the eight fungal genera significantly more abundant in PMM compared with the three other treatments (Fig. 4), the high mean proportion of *Tomentella* spp. was unexpected since it is an ectomycorrhizal genus frequently identified on the root tips of coniferous trees^62,63^. Among the OTUs belonging to *Tomentella* spp., it is interesting to note that the most abundant one was identified as being *T. sublilacina* (63% of the reads). This species has a worldwide distribution, develops ephemeral resupinate basidiocarps buried in the soil, sporulates on decomposing wood and produces thick-walled and spiny spores which facilitates its dispersion via endozoochory and ectozoochory^64,65^.

Despite the reclaimed sites being young (three years old at sampling time), the mycorrhizal community recorded in aspen roots growing in PMM soil featured a relatively high number of OTUs (17) and a composition dissimilar to what was reported in earlier studies that directly analysed the root tips of aspen growing in reclaimed sites^66,67^. The frequency of ectomycorrhizal fungi in post-mining disturbed soils is usually low. For instance, Bois *et al*.^68^ observed only seven ectomycorrhizal fungi on roots of jack pine (*Pinus banksiana*) and hybrid poplar (*Populus deltoides* × *Populus nigra*) grown in soils from old reclaimed sites (13 and 19 years old at sampling time). Regarding root-associated fungi, *Cadophora finlandica* (=*Phialophora finlandia*) was prevalent on aspen roots in PMM. Previous studies found *C. finlandica* to be dominant in burnt and polluted sites^69–71^, suggesting a potential positive effect on stress tolerance of their hosts, particularly in cold environments^72^. Therefore, the relative abundance of this root-associated taxa could be interpreted as a putative stress indicator for their host.

In conclusion, aspen trees that first colonise PMM soil cover reflect the genetic diversity of the surrounding areas but not their belowground microbiome, at least during the first years following reclamation. We also found that PMM soil cover had a limited belowground microbial diversity compared to FFMM and to naturally disturbed soils. The soil microbiome and particularly the mycorrhizal communities are variable both in space and time^73–75^, partly because the root density tends to select for species with different exploration strategies (spores or mycelium)^76^. Therefore, long-term monitoring of the dynamics of above- and belowground diversity is extremely important to ensure that reclaimed plots are on the right ecological trajectory and will return to a state similar to the state of the ecosystem before disruption, and provide similar services.

## Methods

### Study sites and sampling

This study was conducted at an oil sands mine located 75 km north of Fort McMurray, Alberta (57°20′ N, 111°49′ W; Supplementary Fig. S1). The natural forest ecosystem in the region is a boreal mixed wood forest with mesic upland sites consisting of varying mixtures of aspen (*Populus tremuloides* Michx.) and white spruce (*Picea glauca* (Moench) Voss) trees (Beckingham and Archibald 1996). The continental climate has a mean July temperature of 16.8 °C and mean January temperature of −18.8 °C, with mean annual precipitation of 455 mm^77^. The area hosting the two reclamation sites investigated in this study is an 88.6-ha overburden dump constructed of saline-sodic overburden material produced during oil sands mining in 2011. This material was covered with 1.5 m of suitable (i.e. non-saline) overburden and 0.5 m of the reclamation soil cover. FFMM and PMM soil covers (defined in the introduction) were used and are differentiated based on the origin of the organic matter. These soil covers differ in organic matter content, volumetric water content and chemical properties, as shown in Table 1 in Errington and Pinno^78^. In 2014, at the time of sampling, the soil covers differed in the plant community they supported, with FFMM having a greater plant species richness due to the biological legacy of the propagules contained in the forest floor layer^78^ while PMM had a greater density of naturally established aspen^8^. Both reclamation sites were also seeded with barley (*Hordeum vulgare*) in 2011 to control potential erosion and were then planted with white spruce seedlings at the density of 1500 seedlings per ha. For a benchmark comparison, natural sites within a radius of 25 km from the reclamation site were also sampled, including four recent fire-origin sites and four mature forest sites. These sites were dominated by aspen in the overstory canopy with white spruce present as a secondary species in some sites. The soils were all characterised as Gray Luvisols^79^. The four fire-origin sites were all burnt in spring 2011. In 2014, at the time of sampling, there was a post-fire aspen sucker density of approximately 80,000 stems per ha. The age of the mature stands was estimated at between 58 and 70 years old, with an average density of 1450 stems per ha. At each sampling site (FFMM, PMM, Fire and Mature), leaves were sampled on 52 to 109 aspen individuals in late June 2014. Four of these trees at FFMM, PMM and Fire sites were sampled throughout the 2014 growing season in June, July and August to collect root fragments (<0.5 cm in diameter, approximately 5 cm in length) with the surrounding soil. For the Mature site, four aspens were sampled only in July because of access and limited sampling time. From marked aspen trees, the identification of the taproot was used to locate the secondary roots of each tree and a cardinal point was selected for each sampling time. This was done to avoid selecting the same area for further sampling that could reflect unknown disturbance. Secondary roots (about 1 g) and soil samples (about 100 g) were collected at a depth of 8–10 cm and placed into plastic containers. All samples were collected with a sterilized soil knife and gloves and immediately put in a cooler until preparation. Upon return to the laboratory, root samples were washed under running tap water, blot dried, cut up and weighed. Soil samples were homogenized and unwanted content (small stones, roots, etc.) and sifted through a 2 mm sieve.

### Genotyping-by-sequencing of *P. tremuloides* and bioinformatic procedure

DNA for the 343 sampled trees was extracted from dried leaf material using the Nucleospin 96 Plant II kit (Macherey-Nagel, Bethlehem, PA, USA), following the manufacturer’s protocol for vacuum processing with the following modifications: (a) cell lysis using buffers PL2 and PL3 (with PL2 heated for 2 h at 65 °C instead of 30 min) and (b) elution with an in-house Tris-HCl 0.01 mM pH 8.0 buffer. Genotyping by sequencing (GBS) libraries were prepared at the Plateforme d’analyses génomiques of the Institut de biologie intégrative et des systèmes (IBIS - Université Laval, Québec, QC, Canada) according to the procedure described in Poland *et al*.^80^ with *Pst*I and *Msp*I restriction enzymes. Barcoded adapters were ligated to individual DNA and pooled per plate to generate 96-PLEX libraries. The libraries were size selected on a blue pippin 2% agarose cassette and then amplified by PCR. Each amplified library was loaded on three different Ion Torrent™ System P1 v3 chips for sequencing, for a total of 12 chips. The resulting sequences from three chips were merged at the end of the process. Sequencing failed for 23 individuals.

GBS sequences were aligned against the *Populus trichocarpa* reference genome v.3.0^81^ using BWA v0.7.13^82^, together with Samtools v0.1.19^83^ to produce an alignment file for each individual tree. Haplotype variants were then called with Platypus v0.8.1^84^ and filtered using the R package stackr v0.5.3^85^. Eleven more individuals that yielded doubtful results were removed from the data set, leaving 309 individuals for genetic data analyses. Variant calling parameters and filtering are described in the supplementary information file. The detection of clones among samples was conducted by plotting the Gower coefficient of similarity^86^ for each pair of individuals. Pairs of individuals with more than 94% similarity were considered as clone mates (all other pairs shared less than 82% similarity). After all filters, we used the information of nine groups of eight clone mates or more (n = 128) as replicates to estimate the genotyping error rate for each locus, as suggested in Mastretta-Yanes *et al*.^87^, and removed the variants showing an error rate higher than 5%.

### Clonal and genetic diversity of *P. tremuloides*

The index of clonal richness (R), which is the proportion of distinct genotypes present in the sample relative to the number of sampled trees, was calculated according to Dorken and Eckert^88^ and ranges from 0 (all trees are part of the same clone) to 1 (all trees are genetically distinct). Then, one individual per genotype was kept for subsequent genetic data analyses. Observed (*H*_O_) and expected (*H*_E_) heterozygosity estimates were calculated for each site using the R package adegenet v2.0.2^89^. To test for differences in *H*_O_ and *H*_E_ among sites, a one-way ANOVA was performed using the functions *lm* and *anova* in R v3.2.2^90^ with sites as a factor. Global F-statistics and among-site Nei’s pairwise *F*_ST_ statistics^91^ were estimated using the R package hierfstat v0.44-22^92^. The presence of a genetic structure was further investigated using a discriminant analysis of principal components (DAPC) implemented in the R package adegenet. DAPC summarizes the GBS variant data into principal components (PCs) and then performs a discriminant analysis on the retained PCs to describe among-population genetic variation, while overlooking within group genetic variation. We first determined the number of genetic clusters (*k*) useful to describe the data set by running a *k*-means algorithm on all the genetic variation with increasing values of *k* and by computing a Bayesian Information Criterion (BIC) for each *k* as a measure of goodness of fit. DAPC was finally performed using the sites as “predefined” genetic clusters (*k* = 4) with 97 PCs, which corresponds to 70% of the total genetic variance. Unlike Bayesian clustering methods, DAPC does not rely on unverified assumptions about the underlying population genetic model and can describe genetic structure in very large datasets within small computational time.

### Root and soil microbiome: DNA isolation and library preparation

Up to 100 mg of the root sample was transferred to a 2 ml screw cap tube with a metal bead, flash frozen in liquid nitrogen and then ground to a fine powder using a Tissuelyzer II (Qiagen) at 26 Hz for 45 s, twice. DNA was then extracted from the powder using the MoBio PowerSoil DNA Isolation Kit (MoBio Laboratories Inc., Solana Beach, CA, USA). The same kit was used to isolate DNA from soil samples using 250 mg of starting material. DNA was quantified using a Qubit dsDNA HS Assay Kit and Qubit Fluorometer (Life Technologies, Burlington, ON, Canada). Kennedy *et al*.^93^ showed that DNA concentration had more impact on amplification reproducibility than pooling of multiple PCR amplicons, while Schmidt *et al*.^94^ showed a high stochasticity in individual PCR reactions. To limit the potential biases raised by these studies, all sample concentrations were standardised to 5 ng/μl, and each sample was amplified in triplicate. Bacterial communities were targeted using the primer set 341 F (5′-CCTACGGGNGGCWGCAG-3′)/805 R (5′-GACTACHVGGGTATCTAATCC-3′)^95^. The ITS2 region of the fungal ribosomal DNA was amplified using the primer set ITS3_KYO2 (5′-GATGAAGAACGYAGYRAA-3′)^96^/ITS4 (5′-TCCTCCGCTTATTGATATGC-3′)^97^. Primers contained the required Illumina adaptors at the 5′ end of the primer sequences (5′-TCGTCGGCAGCGTCAGATGTGTATAAGAGACAG-3′ for the forward primer and 5′-GTCTCGTGGGCTCGGAGATGTGTATAAGAGACAG-3′ for the reverse primer). The V3-V4 region of the bacterial 16S rRNA gene and the ITS2 region of the fungal rRNA were amplified in 20 μl volumes using 10 μl of HotStarTaq Plus Master Mix (QIAGEN Inc., Germantown, MD, USA), 300 nM of each primer, 7.8 μl of UltraPure™ DNase/RNase-Free Distilled Water (GIBCO, Life Technologies) and 1 μl of gDNA at 5 ng/μl. Thermal cycling conditions were as follows: initial denaturation at 95 °C for 5 min; 30 cycles at 94 °C for 30 s, 50 °C for 30 s, 72 °C for 1 min; and a final elongation at 72 °C for 10 min. PCRs were done on a PTC-200 (MJ Research Inc., Waltham, MA, USA) and products were visualized on GelRed-stained 1% agarose gels using the Chemigenius Bioimaging System (Syngene, Cambridge, UK). PCR triplicates were pooled and cleaned up using 81 μl of magnetic beads solution (Agencourt AMPure XP, Beckman Coulter Life Science, Indianapolis, IN, USA) according to the protocol in Illumina’s “16S Metagenomic Sequencing Library Preparation” guide (Part #15044223 Rev. B). Unique codes were added to each sample by amplifying 5 μl of the purified PCR product with 25 μl of KAPA HIFI HotStart Ready Mix, 300 nM of each Nextera XT Index Primer (Illumina Inc., San Diego, CA, USA) and 10 μl of UltraPure™ DNase/RNase-Free Distilled Water for a total volume of 50 μl. Thermal cycling conditions were as follows: 3 min at 98 °C, 8 cycles of 30 sec at 98 °C, 30 sec at 55 °C, 30 sec at 72 °C, and a final elongation step of 5 min at 72 °C. Indexed amplicons were purified with the magnetic beads as previously described and quantified using a Qubit dsDNA BR Assay Kit and Qubit Fluorometer (Invitrogen, Carlsbad, CA, USA) and combined in an equimolar ratio. Paired-end sequencing (2 × 300 bp) of the 16S and ITS2 pools was carried out on an Illumina MiSeq sequencer at the National Research Council in Montreal. The Illumina and GBS data generated in this study were deposited in the NCBI Sequence Read Archive and are available under the project number PRJNA400551.

### Root and soil microbiome: bioinformatic analyses

Paired-end read assembly, quality filtering (maximum expected error threshold set to 1), length truncation (minimum length set to 400 bp), dereplication, OTUs clustering (similarity threshold set to 97%, singletons excluded) and chimera filtering were performed using usearch v8.0.1623_86linux64^98,99^. The sequencing statistics are provided in supplementary information. The raw number of reads obtained per sample is provided in Supplementary Table S1 and the distribution of raw number of reads per treatment is provided in Supplementary Figure S2. Taxa assignment was done in QIIME v1.9.0-20140227^100^ with the Ribosomal Database Project classifier using a minimum confidence of 0.8. Hierarchical classification of the 16S and ITS2 OTUs were performed against the Greengenes and UNITE (version 7) databases, respectively. Scripts from the Brazilian Microbiome Project were used to convert usearch map files into OTU tables^101^. Consensus reads were aligned with MAFFT v7.017^102^ as implemented in Geneious v8.1.6 (Biomatters Ltd, Auckland, New Zealand). The appropriate alignment algorithm was automatically chosen based on the size of the data set. Columns in the alignment containing at least 95% of gaps were stripped before calculating the phylogenetic tree with FastTree v2.1.5^103^ using the GTR model. Fungal functional groups were predicted using fungal OTUs with a relative abundance (i.e. the percent composition of the reads of an OTU relative to the total number reads observed in the data set) greater than 0.01%. Ecological functions were attributed based on the metadata associated with the BLAST closest matches recovered in the UNITE^104^ and INSDC (www.insdc.org) databases and by cross-referencing the literature (details provided in supplementary information and in Supplementary Tables S2 and S3).

### Alpha and beta diversity analyses

To correct for differences in sequencing depth, the bacterial and fungal data sets were rarefied to the lowest number of reads observed in the libraries in each data set (2424 and 12942 reads, respectively). Within-site (alpha) diversity was calculated using the mean number of OTUs, the chao1 index^105^, the Pielou’s evenness and the Faith’s Phylogenetic diversity index. Rarefaction of OTU tables and the calculation of alpha-diversity indices were done in QIIME. The significance of the differences observed in alpha diversity metrics between sites (PMM, FFMM and Fire only) was assessed separately on root and soil data. Since this analysis included the sampling time as a factor, the site Mature could not be included as it was sampled only in July due to its remote location. To consider the problem of multiplicity associated with testing the null hypothesis of no difference between sites on root and soil data, a Bonferroni correction was applied and each hypothesis was tested at a significance level of $\alpha$ = 0.025 (corrected alpha for each pairwise comparison). A two-way repeated measures ANOVA was performed using the function *lme* in the R package *nlme* v3.1-123^106^ with sites and sampling times as factors. Because no significant difference associated with sampling time was observed, the model was simplified to a one-way repeated measures ANOVA, with sites as a factor. Post-hoc tests were performed using the function *pairwise.t.test* in R package stats v3.1.2.^90^ and a Bonferroni correction was applied to adjust *p*-values for multiple comparisons. Between-site (beta) diversity was first visualized with non-metric multidimensional scaling (NMDS), using the Bray-Curtis dissimilarity distance metric after square root transformation and Wisconsin double standardization. NMDS was calculated with the function *metaMDS* as implemented in the R package *vegan* v2.3–4^107^. The Statistical Analysis of Metagenomic Profiles (STAMP) software package^108^ and the package *ggplot2*^109^ in R were used for visualisation and statistical comparisons between the different sites. To test for differences in bacterial and fungal community composition between the four sites, a one-way analysis of similarity (anosim) was performed on the Bray-Curtis matrices using the software package PRIMER v7^110^ with 9,999 permutations to determine significance. anosim calculates the *R* statistic which ranges from 0 (no community separation) to 1 (dissimilarity between communities). Because the above-mentioned analyses showed that the communities recorded in PMM were the most different from Mature and Fire sites, the sites FFMM/Fire/Mature were combined to perform paired group comparisons with the PMM site in order to find which taxa were the top drivers of the PMM community dissimilarity. More specifically, differences in OTUs relative abundance between the above-mentioned groups of root and soil samples were compared using Welch’s t-test, and false discovery rate (FDR) in multiple testing was controlled by the Storey FDR method^111^. To partition the data sets between abundant and rare OTUs, OTUs which relative abundance is inferior to 0.01% were defined as rare. This cutoff was chosen because OTUs with a relative abundance superior to 0.01% represented ≥90% of the reads in each data set. Hierarchical cluster analyses were performed on the fungal functional profiles determined for each site with the *hclust* function (R package *stats* v3.3.2) and multiscale bootstrap resampling was performed with the *pvclust* function (R package *pvclust* v2.0-0) to calculate approximately unbiased *p*-values for each cluster.

## Electronic supplementary material

Supplementary information

Table S1

Table S2

Table S3
