# Supplementary material for: The impact of reconstructed soils following oil sands exploitation on aspen and its associated belowground microbiome
Source: Sci Rep. 2018 Feb 9;8:2761. doi: 10.1038/s41598-018-20783-6 (PMC5807544; doi:10.1038/s41598-018-20783-6)
Supplement: Supplementary file 1 — Supplementary information [file 41598_2018_20783_MOESM1_ESM.doc]

The impact of reconstructed soils following oil sands exploitation on aspen and its associated belowground microbiome

Franck Stefani^1,2^, Nathalie Isabel^1^, Marie-Josée Morency^1^, Manuel Lamothe^1^, Simon Nadeau^1^, Denis Lachance^1^, Edith H.Y. Li^5^, Charles Greer^3^, Étienne Yergeau^3,4^, Bradley D. Pinno^5^ and Armand Séguin^1*^

^1^Natural Resources Canada, Canadian Forest Service, Laurentian Forestry Centre, Québec, G1V4C7, Canada**.** ^2^Agriculture and Agri-Food Canada, Ottawa, K1A 0C6, Canada. ^3^National Research Council Canada, Energy, Mining and Environment, Montréal, H4P 2R2, Canada**.** ^4^Institut national de la recherche scientifique, Centre INRS-Institut Armand-Frappier, Laval, H7V 1B7, Canada**.** ^5^Natural Resources Canada, Canadian Forest Service, Northern Forestry Centre, Edmonton, T6H 3S5, Canada.

* Correspondence and requests for materials should be addressed to A.S. (email: armand.seguin@canada.ca)

Supplementary information

**Fungal functional groups assignment.** For fungi, only OTUs with a relative abundance > 0.01% were categorised into functional groups. A local database built from the UNITE+INSDC databases (475641 sequences) was queried using blastn ^1^ to match each OTU with its closest ITS sequences.

Based on the metadata associated with conspecific sequences (similarity threshold > 97%) and by cross-referencing the published literature, the following functional groups were recognised: ectomycorrhizal fungi, other mycorrhizal fungi (ericoid, arbutoid, monotropoid and arbuscular), root-associated fungi (OTUs showing a conspecific match with fungal sequences obtained from root tissues, dark septate endophytes, or root endophytes, or with sequencing belonging to the “*H. ericae* aggregate”, i.e. Cadophora-like OTUs), saprotrophs (including fungal pathogens), molds and yeasts (*Saccharomycetes, Tremellomycetes, Zygomycetes*, etc.), fungi with unknown functions (this group includes taxa with mixed trophic status, such as *Morchella* spp., or taxa identified as fungal endophytes in plant stems and leaves, and unknown fungi).

With the exception of OTUs identified as belonging to the *Glomeromycota* (all categorized as “other mycorrhizal fungi”), OTUs showing a similarity threshold < 97% to their closest ITS sequences from the UNITE+INSDC databases or OTUs which genus could not be identified or which the closest sequence was obtained from a strain isolated on a known substrate were categorized into putative functional groups when included in clades containing well-identified functional groups (in the *Sebacina* or *Tomentella* clades, for instance). Also, OTUs were lumped in the “putative” categories when taxa belonging to the same genus were shown in publications to belong to one of the above-mentioned categories. This functional assignment procedure was modified from Clemmensen et al. ^2^ and Phillips et al. ^3^. The functional group assignment was first performed on the fungal data set from roots (Table S1). The fungal functional diversity in soil samples was then determined the same way as for the root samples, with the major exception that the local database built from the UNITE+INSDC databases was upgraded with the root fungal OTUs and the information relative to their functional groups (Table S2). Therefore, soil fungal OTUs were assigned to functional groups based on conspecific matches with root fungal OTUs. Soil fungal OTUs with a pairwise similarity <97% to root fungal OTUs were otherwise assigned into putative functional groups based on their position within clades containing well-identified functional groups, based on the metadata associated with the closest sequences and by cross-referencing the published literature.

**Sequencing statistics.** The MiSeq run yielded a total of 1.9 and 4.9 million raw reads for bacteria and fungi, respectively (Table S1 and Figure S2). Prior to rarefying libraries, the bacterial and fungal data sets contained 6142 OTUs (one million 16S reads) and 4261 OTUs (3.78 million ITS2 reads), respectively. A total of 4919 OTUs (165,512 16S reads) were recovered in the rarefied bacterial data set, while the rarefied fungal dataset included 3394 OTUs (865,114 ITS2 reads). Each rarefied data set was characterised by a long tail of low abundant taxa (Supplementary Figure S3). On average, 20% of the most abundant OTUs represented 90% or more of the reads within each dataset (Supplementary Figure S3). The comparison of Venn diagrams calculated on data sets including or not rare OTUs showed that most of these rare OTUs were specific to each site (Supplementary Figure S4).

**GBS variant calling.** Adapters were removed from the raw GBS sequences using Cutadapt v1.8.1 ^4^, demultiplexed with Sabre v1.0 (<https://github.com/najoshi/sabre>) and verified with FastQC v.0.11.3 (<http://www.bioinformatics.babraham.ac.uk/projects/fastqc/>) prior to alignment.

Haplotype variant calling was done with Platypus v0.8.1 ^5^ using parameters adapted to Ion Torrent sequences (genIndels=0, filterDuplicates=0, maxReadLength=280, minMapQual=17, minBaseQual=20, badReadsThreshold=12) and optimized by visually comparing the different results on challenging alignments (minGoodQualBases=70, rmsmqThreshold=20, hapScoreThreshold=30, trimAdapter=1, maxGOF=20, minReads=2, minFlank=5, scThreshold=0.95, filterVarsByCoverage=0, filteredReadsFrac=0.7, minVarFreq=0.2, mergeClusteredVariants=1, skipDifficultWindows=1, maxVariants=12 and maxReads=3,000,000).

The VCF (Variant Call Format) file produced by Platypus was filtered and analysed with the help of the R package stackr v0.5.3 ^6^. The file originally contained 180,001 variants with a ‘PASS’ status, which was reduced to 55,334 variants after filtering monomorphic loci and those that were not present in all four populations (sites). Then, variant loci showing a heterozygosity over 50% and a global Fis (all individuals) outside the [-0.35 , 0.35] range were excluded since they could be indicative of problems with markers. Only loci presenting less than 30% missing genotypes and a MAF (minor allele frequency) greater than 5% were kept. This left 11,818 high-quality variant loci that were used to investigate possible missing patterns and mixed genomes among individuals. No missing pattern was found by sites or by sequencing plates. However, individuals showing more than 30% missing genotypes (n = 8) were found to have a much lower heterozygosity level than average and three outlier individuals showed a very high heterozygosity level, which is likely the result of operational errors that could have mixed individual DNA; those were excluded from the final data set.

1. Altschul, S. F., Gish, W., Miller, W., Myers, E. W. & Lipman, D. J. Basic local alignment search tool. *J. Mol. Biol.* **215,** 403–410 (1990).

2. Clemmensen, K. E. *et al.* Roots and associated fungi drive long-term carbon sequestration in boreal forest. *Science* **339,** 1615–1618 (2013).

3. Phillips, L. A., Ward, V. & Jones, M. D. Ectomycorrhizal fungi contribute to soil organic matter cycling in sub-boreal forests. *ISME J.* **8,** 699–713 (2014).

4. Martin, M. Cutadapt removes adapter sequences from high-throughput sequencing reads. *EMBnet J.* **17,** pp. 10–12 (2011).

5. Rimmer, A. *et al.* Integrating mapping-, assembly- and haplotype-based approaches for calling variants in clinical sequencing applications. *Nat. Genet.* **46,** 912–918 (2014).

6. Gosselin, T. & Bernatchez, L. *stackr: GBS/RAD Data Exploration, Manipulation and Visualization Using R*. doi:http://dx.doi.org/10.5281/zenodo.154432

Figure S1. Map showing the geographic location of the sampling sites. The map was created with ArcGIS version 10.0 (www.esri.com/software/arcgis) by Roger Brett. Map data: Google, DigitalGlobe and the aerial view is from Google Earth.

**Figure S2.** Distribution of the reads (before rarefaction) per treatment. Error bars represent the standard deviation.


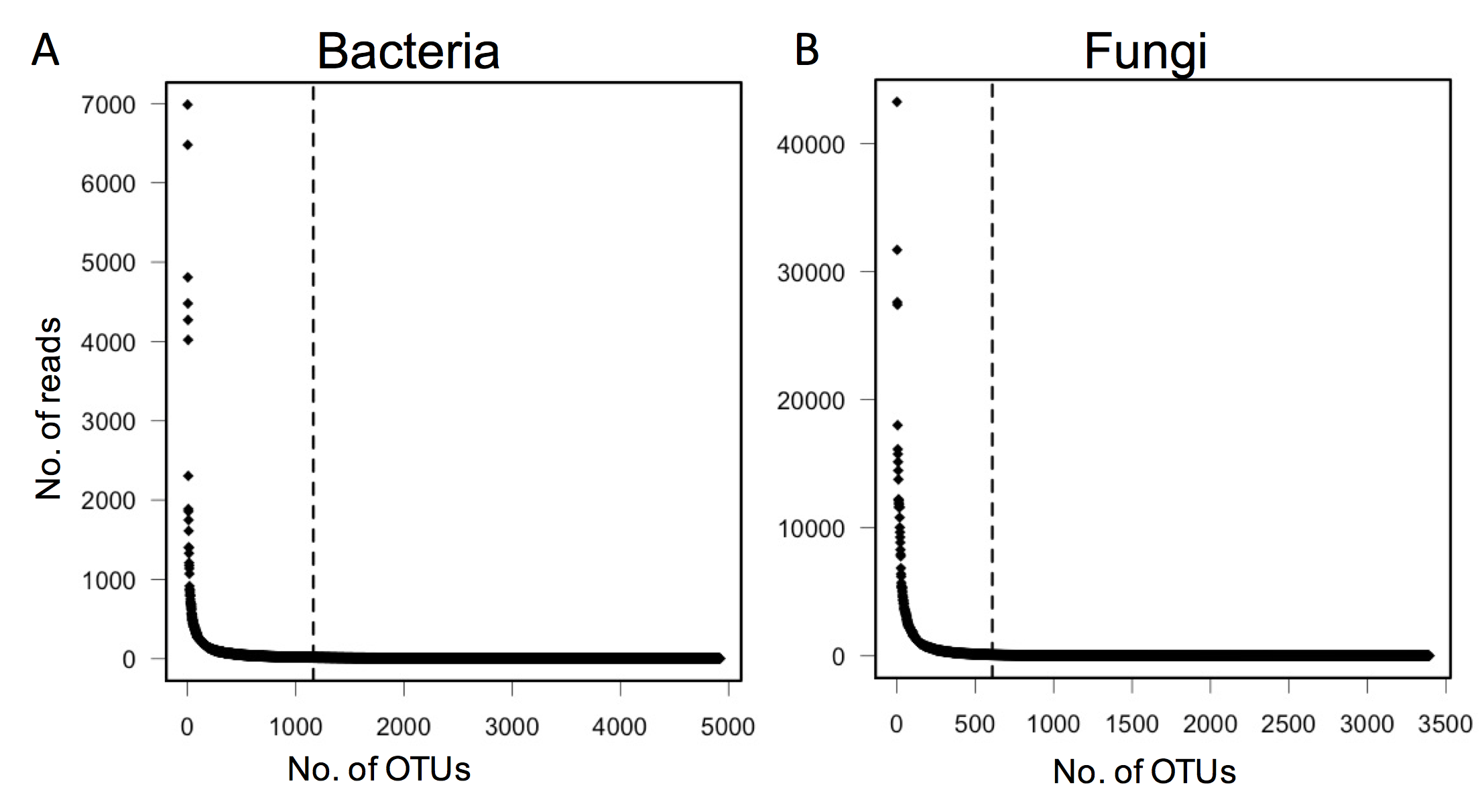


**Figure S3.** Plots of the number of reads versus OTUs, with OTUs ranked according to their respective abundance (rarefied data sets). The dashed lines represent the partition between abundant and rare OTUs, i.e OTUs which relative abundance is inferior to 0.01%. A) 23% of the most abundant OTUs (left of the dashed line) represented 90% of the reads. B) 18% of the most abundant OTUs represented 96% of the reads.


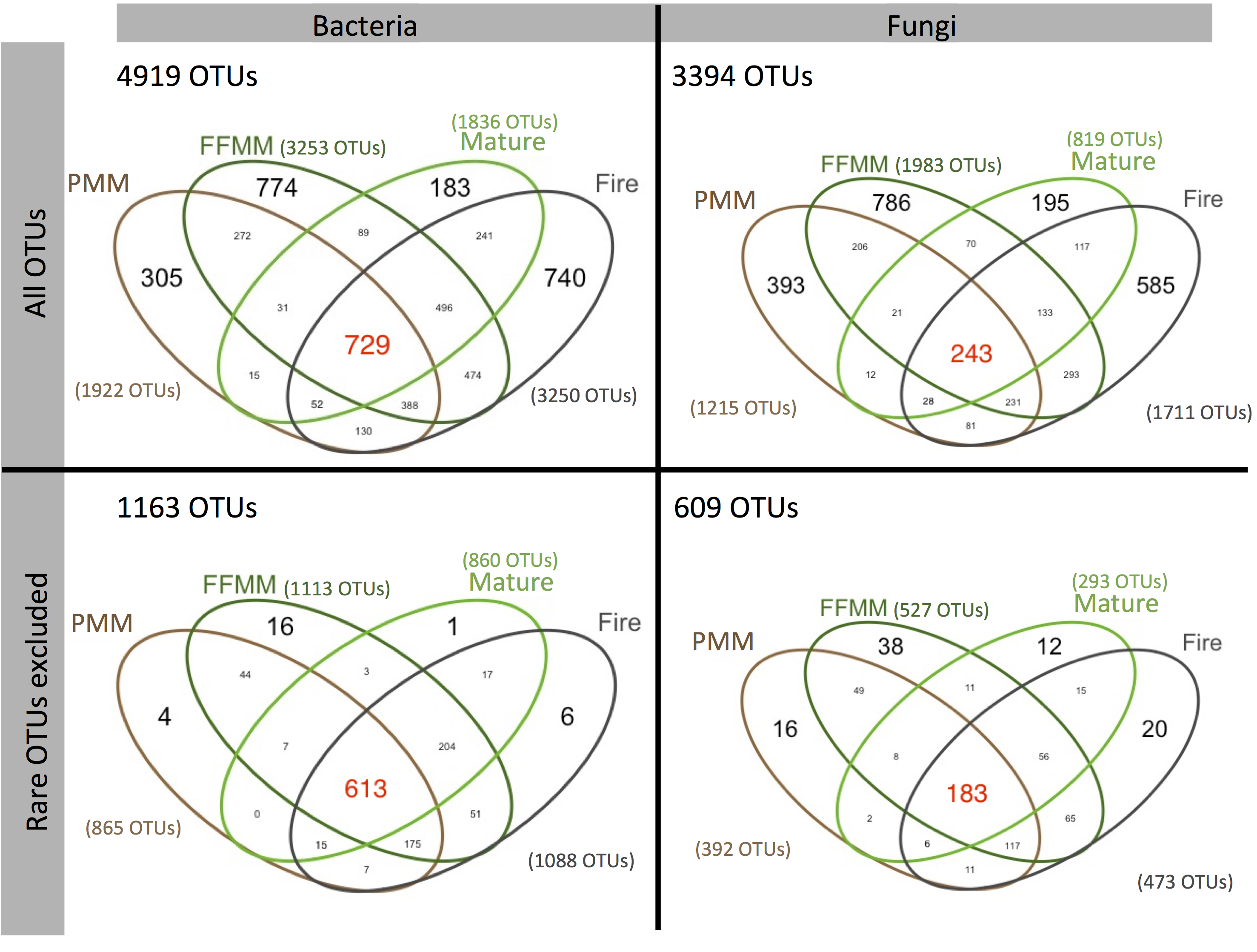


**Figure S4.** Venn diagrams calculated on data sets including or not OTUs defined as rare. See Supplementary Figure S2 for the definition of rare OTUs.
